# Accurate resection of nonpalpable, ultrasonography undetectable breast cancer tumor by preoperative indocyanine green injection using stereotactic mammography: A case report

**DOI:** 10.1016/j.amsu.2022.103965

**Published:** 2022-06-07

**Authors:** Atsushi Muraoka, Masahiko Kobayashi

**Affiliations:** Department of Surgery, Kagawa Rosai Hospital, Kagawa, Japan

**Keywords:** Nonpalpable breast cancer, Localization method, Indocyanine green fluorescence imaging, Stereotactic mammography, Case report, MMG, mammography, US, ultrasonography, MRI, magnetic resonance imaging, SLNB, sentinel lymph node biopsy, ICG, indocyanine green

## Abstract

**Introduction:**

and importance: Tumor localization is vital in the surgical management of nonpalpable breast cancer. Various localization methods exist, each with their own disadvantages. Therefore, we need to investigate the optimal method of diagnosis for this condition.

**Case presentation:**

A 66-year-old woman presented to our facility with a microcalcification detected on screening mammography (MMG). The lesion was neither palpable nor detectable on ultrasonography (US). Three-dimensional stereotactic biopsy using MMG revealed ductal carcinoma in situ. The precise tumor location was needed to perform breast-conserving surgery.

**Clinical discussion:**

Our hospital did not have radioisotope imaging; hence, wire placement would have been difficult for this lesion location. To aid in localization, indocyanine green (ICG) and fluorescence imaging were used. ICG was injected preoperatively using stereotactic MMG, which enabled clear visualization of the lesion. Then, an accurate resection was performed. The patient was discharged without any complications 2 days after surgery.

**Conclusion:**

The findings of this case report suggest that stereotactic MMG-guided ICG can be useful in localizing breast cancer tumors that are nonpalpable and undetectable by US.

## Highlights

• ・The incidence of nonpalpable breast cancer has increased.
• ・Precise tumor localization is crucial for surgically managing such breast cancers.
• ・Fluorescence imaging localization using stereotactile mammography is valid for ultrasonography undetectable breast cancer.

## 1 Introduction

The incidence of nonpalpable breast cancer has increased with the adoption of population screening programs [1]. Ductal carcinoma in situ is often detected as microcalcification on mammography (MMG). However, small, calcified lesions cannot be visualized by ultrasonography (US). In such cases, stereotactic biopsy using MMG is commonly indicated for diagnosis. Surgical resection is the gold standard treatment for malignant lesions, regardless of tumor size. Accurate localization is crucial for optimal surgical outcomes to avoid unnecessary surgical resection and improve cosmetic outcomes. Although wire-guided localization is commonly used, it has some disadvantages such as wire rupture, migration, or infection [2,3]. We herein describe a unique localization method using fluorescence imaging followed by successful surgical resection for a nonpalpable, US undetectable breast cancer.

This case report has been reported in line with the SCARE criteria [ [4]].

## 2 Presentation of case

**Fig. 1:**
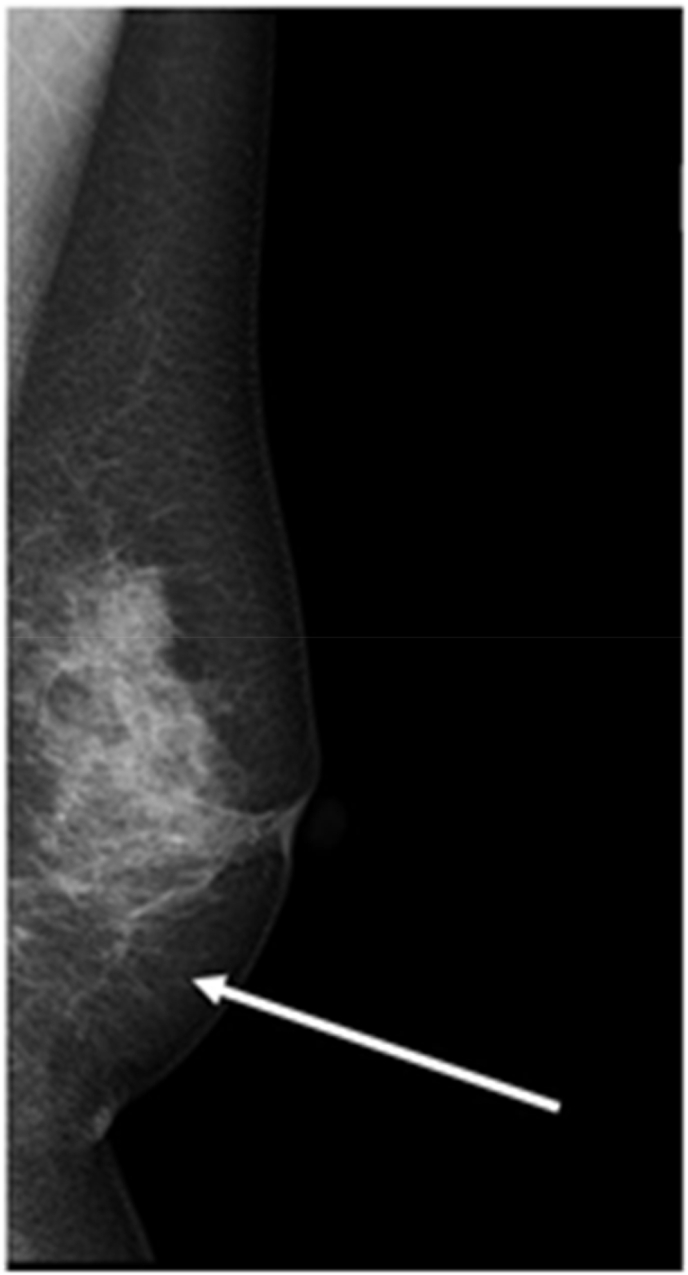
Mammography demonstrating microcalcifications at the lower and medial site (arrow).

**Fig. 2:**
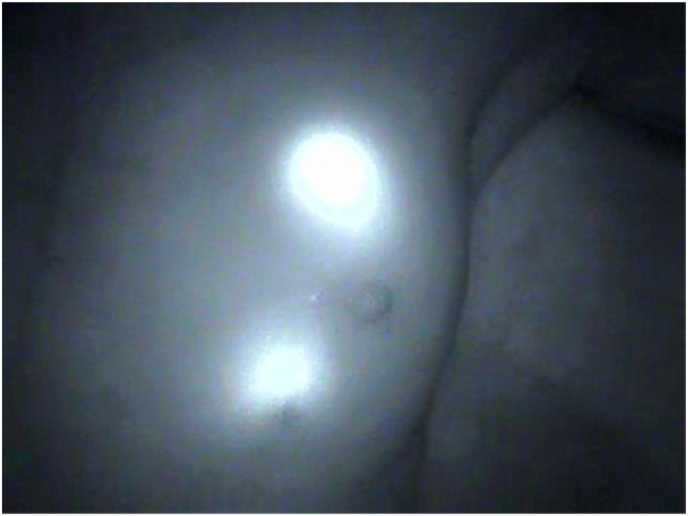
Fluorescence imaging showing the inserted needle (upper) and injected tumor (lower).

A 66-year-old woman presented with small microcalcifications detected on routine screening MMG (Fig. 1). She had no significant past medical and family history and no allergies including that for iodine. The lesion was nonpalpable and undetectable on US. Three-dimensional stereotactic biopsy using MMG identified the calcified site as ductal carcinoma in situ. The patient opted for breast-conserving surgery. Excessive removal of breast tissue was likely to lead to deformation and a poor cosmetic outcome because the lesion was at the lower and medial side of the breast. Hence, the precise tumor location was needed for surgery. Common methods such as wire-guided or dye-guided localization require wider resections. Therefore, a more sensitive localization method was needed for the small lesion in this patient. Fluorescence imaging was used for tumor localization as a part of sentinel node navigation. The calcified lesion was detected using the diagnostic stereotactic MMG procedure performed by a radiologist. A 16-gauge needle was inserted under local anesthesia and 0.5 mL of indocyanine green (ICG) solution (Diagnogreen, Daiichi pharmaceutical, Tokyo, Japan) mixed with lidocaine jelly (Xylocaine Jelly, Sandoz Pharma K.K., Tokyo, Japan) (ICG amount:5mg) was injected into the calcified lesion without massage. Surgery was performed approximately 1 h after the injection. A fluorescence camera system (HyperEye Medical System, Mizuho, Japan) clearly visualized both the inserted needle and the injected lesion (Fig. 2).

**Fig. 3:**
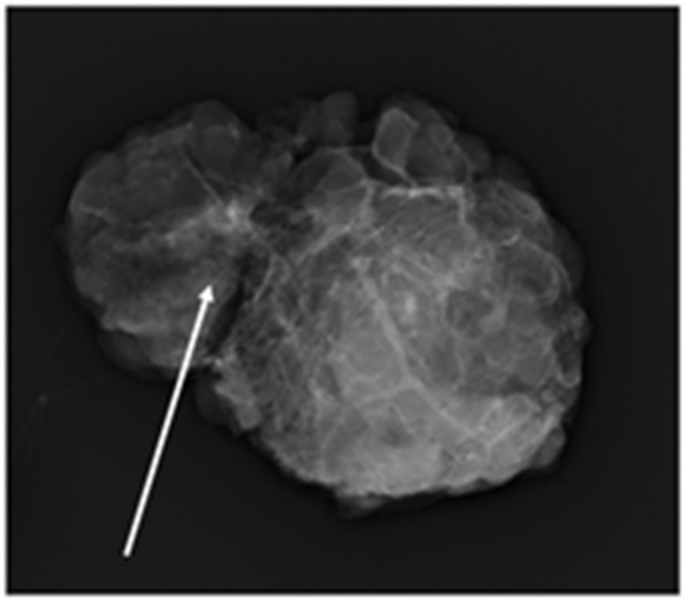
Resected specimen using mammography showing the inclusion of all microcalcifications.

**Fig. 4:**
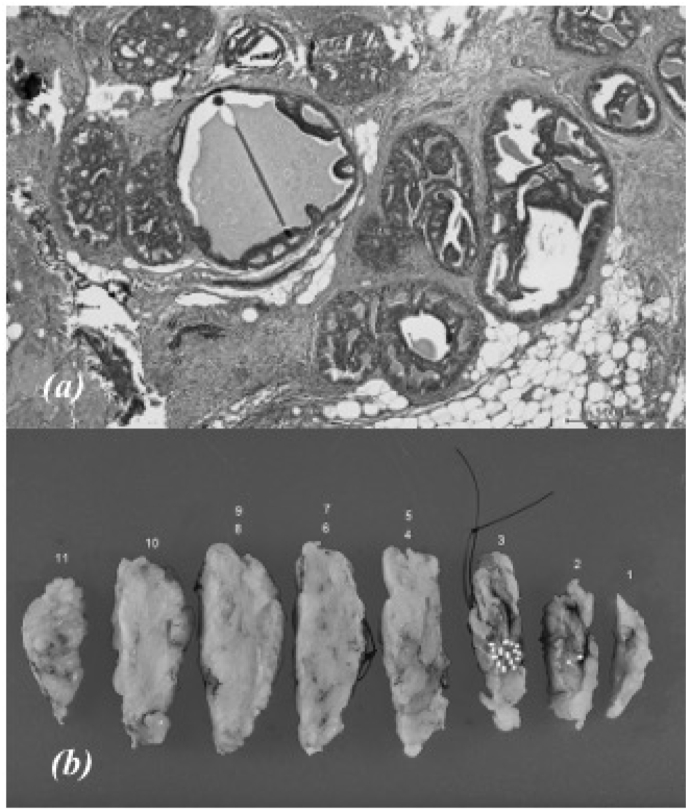
(a) Photomicrography showing ductal carcinoma in situ (H&E staining; × 80) (b) Mapping of resected specimen demonstrating negative surgical margins (white dots: cancerous lesions).

The extent of resection was determined via preoperative magnetic resonance imaging (MRI). The resected specimen was grossly assessed to ensure complete removal of the microcalcifications seen on MMG (Fig. 3), and frozen section analysis confirmed clear surgical margins. The patient had an uneventful recovery and was discharged 2 days after surgery. Pathological evaluation guided a diagnosis of ductal carcinoma in situ (van Nuys classification: Group 1), and negative cancerous margins were confirmed (Fig. 4a and b). The patient is in good health without recurrence.

## 3 Discussion

The incidence of nonpalpable breast cancer has been increasing because of the widespread implementation of breast cancer screening. Early detection and treatment of nonpalpable breast malignancies decrease morbidity and mortality [1]. Microcalcification is the most common sign of nonpalpable breast cancer and can be the only manifestation of the disease. Once microcalcifications are diagnosed as malignant, surgical management is recommended. Accurate tumor localization is necessary to optimize surgical outcomes in terms of both good margins and cosmetic results. To this end, several localization techniques have been described [5]. Wire-guided localization is currently the most common localization technique; however, it has several risks, such as wire rupture, migration, or infection. Furthermore, it can provide misleading results in cases of dense breast tissue. Carbon marking has been used as a safer alternative; however, it can lead to imprecise localization and skin tattooing. Radio-guided occult lesion localization using radioactive particles has also become accepted widely [6]. This technique localizes the cancerous site as well as the sentinel node but requires technical expertise and is costly. Furthermore, radiation exposure is inevitable. Intraoperative US-guided localization is simple and useful, but only if US is able to detect the lesions [7].

Sentinel lymph node biopsy (SLNB) is conventionally used to investigate the axillary lymph node status. Although the blue dye-radioisotope method is well adopted in SLNB, recent reports demonstrate that fluorescence-guided imaging has equivalent efficacy. In this case, the lesion was small, nonpalpable, and not detected by US. A precise localization was needed for her operation. Our hospital had no radioisotope imaging, and preoperative wire placement was deemed difficult, with high risk of dislocation. Therefore, fluorescence imaging was used. Preoperative injection of ICG was performed at the radiology department using MMG. Unlike intraoperative ICG injection by US guidance, time management between ICG injection and operation is mandatory because of dye diffusion. Hence, lidocaine jelly was mixed with ICG. The time elapsed between the injection and operation in our case was 1 h. Dye diffusion did not occur; however, further research is needed concerning waiting time, dye dose injected, and prevention of dye diffusion. This localization method using ICG marking guided by stereotactic MMG has not been reported previously. Additionally, this procedure enables concurrent sentinel node localization. The good outcome of ICG-guided localization by US was reported recently [8]. This fluorescence method allows all device-guided localization for nonpalpable breast lesions including MRI. Although this procedure relies on the availability of fluorescence imaging, this is also the case for sentinel lymph node navigation using ICG. If this fluorescence localization method is widely adopted, it has the potential to provide more cost-effective and accurate surgical localization and resection, as well as being comparatively simple to perform.

## 4 Conclusion

In conclusion, fluorescence imaging guided by stereotactic MMG can be used to localize a nonpalpable, US undetectable carcinoma. This is the first case report documenting such as use, and this may be a convenient and valuable localization option for nonpalpable, US undetectable breast cancer.

## Consent of patient

Written informed consent was obtained from the patient for publication of this case report and accompanying images. A copy of the written consent is available for review by the Editor-in-Chief of this journal on request. The diagnostic and management approach were explained to the patient, including benefits and possible complications. The patient provided informed consent for all procedures.

## Patient perspective

The procedure of surgery was explained to the patient with all advantages and possible complications. She agreed to the procedure and provided informed consent.

## Provenance and peer review

Not commissioned, externally peer-review.

## Sources of funding

The authors have no sponsors.

## Ethical approval

The requirement for ethical approval was waived by the Institutional Review Board because this was a case report.

## Research registration

1. Name of the registry: Not applicable
2. Unique identifying number or registration ID: Not applicable
3. Hyperlink to your specific registration (must be publicly accessible and will be checked): Not applicable

## Guarantor

Atsushi Muraoka.

## Annals of medicine and surgery

The following information is required for submission. Please note that failure to respond to these questions/statements will mean your submission will be returned. If you have nothing to declare in any of these categories then this should be stated.

## Ethical approval

The requirement for ethical approval was waived by the Institutional Review Board because this was a case report.

## Please state any sources of funding for your research

The authors have no sponsors.

## Author contribution

Atsushi Muraoka drafted the manuscript, performed the operation and Masahiko Kobayashi reviewed the manuscript.

## Please state any conflicts of interest

None declared.

## Registration of research studies

1. Name of the registry:

None.

2. Unique Identifying number or registration ID:

None.

3. Hyperlink to your specific registration (must be publicly accessible and will be checked):

## Guarantor

Atsushi Muraoka.

## Consent

The procedure of surgery was explained to the patient with all advantages and possible complications. She agreed to the procedure and provided informed consent.

Written informed consent was obtained from the patient for publication of this case report and accompanying images. A copy of the written consent is available for review by the Editor-in-Chief of this journal on request.

## Declaration of competing interest

None declared.
